# Role of Enhanced Recovery after Surgery (ERAS) Protocol in the Management of Elderly Patients with Glioblastoma

**DOI:** 10.3390/jcm12186032

**Published:** 2023-09-18

**Authors:** Ismail Zaed, Francesco Marchi, Davide Milani, Ivan Cabrilo, Andrea Cardia

**Affiliations:** Department of Neurosurgery, Neurocenter of South Switzerland, EOC, via Tesserete 44, 6900 Lugano, Switzerland

**Keywords:** ERAS, enhanced recovery after surgery, elderly, neurosurgery, glioblastoma

## Abstract

Objective: Among the already difficult management of neuro-oncological patients, the elderly population remains vulnerable. Because of the pathology and the comorbidities, they present a significantly higher rate of medical issues related to surgical management. Despite this, the surgical option, if feasible, remains the gold standard in these patients, and an Enhanced Recovery After Surgery (ERAS) protocol could improve the postoperative safety of the patients. With this purpose, we prepared this study with the aim of defining the postoperative hospital length of stay (LOS), but also of evaluating the postoperative morbidity, perioperative complications, and postoperative pain scores. Methods: This was a retrospective, single-cohort study performed at an academic hospital (Department of Neurosurgery, Neurocenter of South Switzerland, Switzerland) on elderly patients who underwent craniotomy for glioblastoma. Patients were enrolled in a novel ERAS protocol from January 2022 to December 2022. Since this is a feasibility study and a direct comparison was not possible, we used a historical cohort of elderly patients who had undergone elective craniotomy surgery for glioblastoma as a control group. Results: A total of 19 patients treated in our center for glioblastoma multiforme (GBM) who were aged over 75 years were included in this study. Among those, seven were newly recruited patients included in the ERAS protocol, while the remaining twelve were part of a historical cohort of previously treated patients. From a statistical point of view, the two cohorts were comparable in terms of baseline demographics. In the follow-up, it was shown that in the ERAS group, there was a reduction in the use of opioids after the surgical procedures that could be seen at 30 days (36.2% vs. 71.7%, *p* < 0.001), but also at 3 months, after surgery (33.0% vs. 80.0%, *p* < 0.001). A significant difference has also been documented in terms of mobilization and ambulation: compared to the historical cohort, in the ERAS group, there was a higher rate of mobilization (60.0% vs. 10.0%, *p* < 0.001), but also of ambulation (36.1% vs. 10.0%, *p* < 0.001). Conclusions: The ERAS protocol for the management of glioblastoma in elderly patients seems to be an effective option for reducing LOS in the hospital, as well as for reducing the number of days spent in the ICU, improving the general recovery of the patient, and reducing the costs associated with hospitalization.

## 1. Introduction

Enhanced Recovery After Surgery (ERAS) is now known to be a set of strategies and clinical programs that aim to improve the short-term outcome after a surgical procedure, with a secondary effect on patient comfort, cost-effectiveness, and overall perioperative healthcare utilization [1].

Despite being used only recently in the neurosurgical field, the first ERAS concepts were proposed in the 1990s [1,2]. At last, in 2001, there was a formal introduction of the concept by the ERAS study group [3], which resulted in the publication of an article regarding the surgical management of rectal oncological lesions that shifted the paradigm of care [4].

Currently, the ERAS protocols aim to achieve the purpose of improving surgical outcomes through a selected set of interventions, also known as elements, that include a series of iterative actions involving a multidisciplinary team (surgeons, anesthesiologists, nurses, and patients) [5,6,7,8,9,10,11]. In recent years, several studies have demonstrated that, through the systematic use of such innovative protocols, it was possible to achieve a significant reduction in different aspects of constant worry in neurosurgical care, such as the length of stay (LOS), the management of postoperative pain, and the postoperative complications and economic costs related to the hospitalization [6,7,8,9,10]. Although ERAS protocols, or at least some of their core elements, have been widely adopted in different surgical specialties, the use of ERAS in neurosurgery has only recently begun. There was originally greater interest in spine surgery, but more recently in cranial surgery as well [6,7,12].

Until now, there has been little evidence regarding the use of such a protocol in cranial neurosurgery, especially concerning its applicability in the elderly population. To fill this gap in our knowledge, our aim was to present a feasibility study on the use of ERAS strategies for craniotomy procedures in elderly oncological patients with glioblastoma.

## 2. Material and Method

### 2.1. Patient Selection 

In this study, we included all the older patients (>75 years old) who had been diagnosed with a single primary intracranial lesion suspected of high-grade glioma (HGG) who were medically and surgically eligible for surgical resection. All the patients were included in a one-year period (January 2022 to December 2022). All patients were operated on at the Neurocenter of South Switzerland (Lugano, Switzerland).

In order to avoid possible biases, several exclusion criteria were adopted: all patients that required immediate care, including those who had been diagnosed with pathologies requiring surgery in an emergency setting or those with preoperative disturbances of consciousness, or diseases that could impact postoperative recovery, were excluded. Also, all patients who were unable to follow the instructions of ERAS elements were excluded. 

Given the requirement for active patient participation, we decided to propose a feasibility study on a small cohort to determine whether it was applicable to our department and if it presented advantages to patients. The statistical significance of the protocol implementation was assessed by comparison with a historical cohort.

### 2.2. ERAS Protocol and Conventional Care 

In order to establish an effective workflow, we set up a neurosurgical ERAS group including all the personnel involved in the preoperative, intraoperative, and postoperative management of patients scheduled for elective craniotomy procedures for high-grade glioma (among which were neurosurgeons, anesthesiologists, nurse anesthetists, floor nurses, pre-admission staff, dieticians, physiotherapists, social workers, and occupational therapists). This multidisciplinary group focused its efforts on creating, testing, and applying the neurosurgical ERAS protocol outlined in this study. 

The ERAS elements included in this protocol were based on previous experiences in cranial, spinal, abdominal, and pelvic surgery, according to a review of the related scientific literature [12,13,14]. 

As is similar to most ERAS protocols, we proposed one with three main sections, consisting of preoperative, intraoperative, and postoperative phases. 

In the first phase, there was complete anesthesiologic and surgical counseling to better define all the elements that affect postoperative recovery, among which preoperative functional status evaluation, smoking and alcohol usage status, evaluation of antithrombotic prophylaxis, and nutritional assessment were included. In the second phase, the intraoperative phase, there was great attention paid to using a surgical approach as non-invasive as possible for the craniotomy procedures. However, due to antalgic actions, such as the use of scalp block and the administration of non-opioid drugs, there was also an anesthesiologic interest in and focus on avoiding fluid imbalance and hypothermia. After the surgical procedure, closure was performed using absorbable skin sutures. In the third phase, there was an effort by several components of the ERAS team, including an anesthesiologist, nurses, and a physiotherapist, to encourage early mobilization from the bed, a personalized postoperative diet, and an early neuropsychological assessment. This was coordinated by a senior resident under the supervision of an attending.

Once the patients were enrolled in this study, the patients’ medical records were strictly followed by the ERAS Neurosurgical Group, with a member responsible for each element of the ERAS protocol. All the elements of the ERAS protocols are summarized in Table 1. Concerning the historical cohort, there were no strict guidelines regarding their management, and they were treated with the conventional forms of care under individual practice patterns.

### 2.3. Outcome Measurements 

The demographic information (age, gender), nutritional details before surgery (total body weight, BMI), preoperative comorbidities (classified by the American Society of Anesthesiologists (ASA)), and patient-specific ailments (such as smoking, diabetes, hypertension, hypercholesterolemia, etc.) of the patients were evaluated and documented upon admission.

The primary endpoint of this investigation was centered on the length of postoperative hospital stay, denoting the number of days between the procedure and the discharge date within the initial hospitalization period. The complete hospitalization duration and the rate of readmissions within 30 days were also registered. The cumulative hospital stay duration encompassed both the initial stay and any subsequent readmissions.

Secondary objectives encompassed postoperative morbidity, surgical complications (like surgical site infections, intracranial infections, epilepsy, and hemorrhages), non-surgical issues (including respiratory, cardiovascular, gastrointestinal, and urinary tract problems, as well as venous thromboembolism), status of functional recovery (measured by discharge and 30-day Karnofsky Performance Status (KPS) score), and patient contentment assessments.

### 2.4. Discharge Criteria 

As was one of the objectives of the newly established protocol, patients were discharged as soon as clinically possible. The decision to discharge a patient has been always made by a consensus between two senior attendings and a senior resident who have been instructed to follow the discharging criteria. Several criteria were used to determine whether a patient was dischargeable: a reasonable VAS score with oral antalgic medication, no signs of clinical postoperative infection, intake of solid food, independent mobility, and safe disposition at home. 

### 2.5. Ethical Approval

This study was conducted according to the Ethical Principles for Medical Research Involving Human Subjects, stated in 2004, and the further revisions made in 2008 and 2013 of the Declaration of Helsinki. 

Because of the nature of the proposed study, no approval from the ethics committee was needed.

### 2.6. Statistical Analysis 

All pertinent patient characteristics were compared using descriptive statistics. Student’s *t*-test was used to search for group differences in continuous data with a normal distribution. The Mann–Whitney U-test was used to analyze the data, since there was no normal distribution. Fisher’s exact test or the chi-square test were used to analyze the readmission, complication, and fatality rates. Statistical significance was defined as a *p*-value below 0.05.

Each statistical analysis was conducted (version 19, IBM Corp.) using SPSS (version 19, IBM Corp., Armonk, NY, USA).

## 3. Results

A total of 19 patients were eligible to be included in the study. The baseline demographic characteristics were similar between the two groups, with no statistically significant differences, as summarized in Table 2. Because of this, we compared the two groups from a statistical point of view. Of the 19 patients, 7 (36.8%) were from the ERAS group, whereas the remaining 12 (63.2%) were from the historical cohort. 

No significant differences were found in the number of factors that could affect the postoperative outcome, such as smoking status, the presence of diabetes, hypertension, COPD, and substance abuse disorders.

In Table 3, we summarize the median surgery duration, intraoperative fluids, urine output, and blood loss; these are all factors known to affect the immediate postoperative outcomes of neurosurgical patients. All the postoperative factors were comparable between the two groups; there was, however, a difference in the amount of intraoperatively administered crystalloids, since this was significantly higher in the control group. 

In Table 4, we summarized the preoperative factors related to the objectives of the study. No significant differences were found in 30-day all-cause readmission rates or the reoperation rates for any indication within 30 days; there were, however, statistical differences in terms of days spent in the neuro-ICU (*p* = 0.03) and in the number of days spent in the surgical ward (6.3 days vs. 10.5 days, *p* < 0.001). Several factors affected the length of stay in the ICU, in particular fever, hyperglycemia, and dysnatremia, as well as a transient case of lower nerve dysfunction and a postoperative case needing an EVD.

The reduction in the total number of days spent in our institution was mainly due to the reduction in postoperative pain and the strict organization of the demission with the help of social workers.

## 4. Discussion

Despite its vast use in other surgical specialties and in spine surgery, up until now, the scientific evidence for the use of ERAS protocols is limited [15]. This is especially a topic of discussion regarding elderly patients, among whom a larger number of comorbidities are to be considered. 

In this feasibility study, we considered only elderly patients (>75 years) treated surgically for high-grade gliomas. Thanks to the comparison with a historical cohort of statistically comparable patients, we were able to prove that the implementation of an ERAS protocol with multidisciplinary elements from different specialties (neurosurgery, anesthesiology, physiotherapy, etc.) is not only a feasible option, but will also help in reducing the time spent in the ICU, along with the related risks of infections and costs. This first study was also able to show that the implementation of such protocols helps in improving the decreased use of painkillers, in particular opioids, due to the improvement of pain control with early mobilization. 

When our study was first conceived and proposed, there was little to no evidence that ERAS protocols could be applied for elderly HGGs patients. In previous experiences, in particular those by Hagan et al., there have been guidelines proposed based on non-neurosurgical evidence, which presented interesting elements but needed more evidence before being applied to craniotomy patients [16]. 

Previous studies in the field have suggested that ERAS protocols, but also some isolated elements, could be of benefit in terms of LOS and clinical outcome [17]. It was until very recently, in 2019, that there was the publication of the first significant evidence for this. Wang et al. reported their experience with 140 patients undergoing surgical intervention for cerebral tumors [18]. Through a series of articles discussing the same patients, they also reported other clinical advantages of ERAS protocols, in particular, glucose homeostasis and an improved rate of patient satisfaction [19]. In our experience, as well as in other similar experiences, different ERAS elements were based on previous recommendations. 

As in our previous experience with spinal neurosurgical procedures, we maintained an aggressive attitude toward analgesia both in the intraoperative and the postoperative phases. In particular, as has been also suggested by Elayat et al. [15], we proceeded with the administration of perioperative flupiritine and bilateral complete scalp blocks. Through these actions, it was possible to achieve a lower rate of postoperative pain that, in the majority of cases, was possible to control with non-opioid painkillers, especially after the first days. Contrary to previous experiences of other authors, we did not give relevance to the ways in which stitching was performed on the surgical wounds. The conventional protocol, consisting of the use of surgical clips on the epidermis, was applied. In order to create the ERAS protocol implemented in this study, previous neurosurgical experiences, especially in case series involving the spine, were taken in consideration. 

An important result that we obtained was a significant reduction in the proportion of patients requiring hospitalization in the Intensive Care Unit for more than 2 days; this has also had positive effects on the duration of the hospital stay, since the length was statistically reduced. This led to important benefits not only for the overall outcome of the patients, with the increase in their satisfaction rates, but also in terms of cost-effectiveness. The factors affecting LOS after surgical procedures are known to vary widely [20,21]. 

As has often been shown by previous studies, scalp blocks provide better results in terms of inflammatory response and better pain control in the case of elective craniotomy compared to incision site infiltration with local anesthetics [14]. Postoperative pain management was also improved by the use of other non-opioid agents that act at the central level, such as flupiritine [22]. 

### 4.1. Limitations of the Study 

Despite the authors’ best efforts, the present study has several limitations. The main limitation is due to the nature of the study itself, since it was retrospective and monocentric. Because of that, there was no possible randomization, which could be responsible for potential bias. It should be pointed out that another limitation exists due not only to the nature of the study, but also to the number of patients.

However, since this was a feasibility study, the cohort included in the study was relatively small, but could be enough to provide sufficient scientific evidence. As has been stated, the control group used in this comparative study was the retrospective series present in our institution. Because of that, the data were gathered in a retrospective fashion. 

### 4.2. Future Directions 

Recent years have been characterized by an increasing interest in this field, and the efforts are supported by encouraging results. Future studies should focus on defining whether compliance varies by age and whether it is associated with different outcomes. Another topic that should be addressed is the long-term outcomes associated with ERAS protocols in the elderly community. We also hope that our study will be replicated in the future to confirm the results of our study through a prospective, randomized clinical trial. 

## 5. Conclusions

ERAS protocols are multimodal, interdisciplinary practices designed to involve every stage of the surgical journey and improve a variety of outcomes. 

The ERAS protocol for the management of glioblastoma in elderly patients seems to be an effective option for reducing LOS in the hospital, but also reducing the days spent in the ICU, thus improving the general recovery of the patient and reducing the costs associated with the hospitalization.

## Figures and Tables

**Table 1:** ERAS checklist applied in this feasibility study.

Element	Action
Preoperative	
Patient education	Exhaustive explanation of benefits of abstaining from alcohol and smoking, but also of early ambulation and discharge from ICU.
Preemptive analgesia	Flupiritine maleate (100 mg) is give to all patients the 2 h before surgery
Nutrition optimization	Preoperative maltodextrin 100 mL is given 2 h before surgery
Diabetes management	Measured and maanged in all patients
**Intraoperative**	
Pain management	scalp blocks with bupivacaine 0.25% is given after the inductions
Normothermia	Ensured with warm IV fluids and forced air warmers and body tempterature monitored
Intraoperative IV fluid therapy	Goal-directed approach
Surgical technique	As minimally invasive as allowed
Blake drainage tube	only used in special circumstances
**Postoperative**	
Pain management	Avoidance of opiods
Mobilization	Early mobilization
Foley’s catheter	Removed the first day, after mobilization
Postoperative alimentation	Oral sips encouraged immediately 2 h after surgery
DVT prophylaxis	DVT pumps immediately postoperatively. Chemical prophylaxis considered only in plegic patients

**Table 2:** Demographic data of patients included in the study.

	ERAS Group	Control Group
**N patients**	7	12
**Gender**		
Male	2	5
Female	5	7
**Average age**	77.1 (75–81)	76.4 (75–83)
**Smoking status**		
Current	1	3
Former	3	2
Never	3	7
**Diabetes**	2	3
**Hypertension**	5	7
**COPD**	0	0
**Substance abuse disorder**		
Yes	0	0
No	7	12

**Table 3:** Data related to the intraoperative time.

	ERAS Group	Control Group	*p* Value
**Median length of procedure (minutes)**	253.14 ± 72.03	283 + 91.83	0.23
**Median blood loss during surgery (mL)**	824.57 + 518.01	711.43 + 463.06	0.14
**Median intraop crystalloid (mL)**	2298.57 + 552.47	2375.71 + 777.28	0.02

**Table 4:** Postoperative outcome of the patients included in the study.

	ERAS Group	Control Group	*p* Value
**Number of patients staying more than 48 h in ICU (n)**	1	5	0.03
**Median total hospital LOS from admission to discharge in days**	6.3	10.5	0.001
**30-day all-cause readmission rate, no. (%)**	0	1	NS
**Reoperation rate for any indication w/in 30 days, no. (%)**	0	0	NS

## Data Availability

Available upon request.
